# A nomogram for predicting malnutrition risk in patients with chronic heart failure and correlation study between GHRL, MSTN, CRP, Hs-CRP

**DOI:** 10.1186/s12872-025-04985-1

**Published:** 2025-08-14

**Authors:** Yuling Zha, Chengshuai Liu, Yuping Zhou, Miao Kong, Jinlei Liu, Lu Jing, Hailati Gulimila

**Affiliations:** 1 Eye Hospital, China Academy of Chinese Medicine Sciences, Beijing, 100040 China; 2 Beijing University of Chinese Medicine, Beijing, 100029 China; 3 Guang’anmen Hospital, China Academy of Chinese Medical Sciences, Beijing, 100053 China; 4 Yili Prefecture Traditional Chinese Medicine Hospital, Xinjiang, 83500 China

**Keywords:** Chronic heart failure, Malnutrition, Ghrelin, Myostatin, Nomogram

## Abstract

**Objective:**

This study aimed to construct a nomogram to identify risk factors for malnutrition in patients with chronic heart failure (CHF) and to explore the correlation between Ghrelin (GHRL), Myostatin (MSTN), C-reactive protein (CRP) and High-sensitivity C-reactive protein (Hs-CRP) to further elucidate the potential pathophysiological mechanisms linking malnutrition/sarcopenia and inflammation.

**Methods:**

A total of 128 patients with congestive heart failure (CHF) admitted to the Cardiology Department of Guang’anmen Hospital, China Academy of Chinese Medical Sciences, between February 2022 and February 2023, were included in the study. Based on their MNA-SF scale scores, the patients were classified into two groups: the malnutrition group (107 patients) and the non-malnutrition group (21 patients). Univariate and multivariate logistic regression analyses were performed to identify risk factors for malnutrition in CHF patients, which facilitated the development of a nomogram. Correlation analysis was also conducted to explore the relationships between GHRL, MSTN, CRP, and Hs-CRP.

**Results:**

Logistic regression analysis identified age, right upper limb diameter, simplified anorexia scale score, and MSTN as significant risk factors for malnutrition in CHF patients (*P* < 0.05). The nomogram exhibited strong discriminative power during internal validation, with an AUC of 0.917 (95% CI: 0.8439–0.990), a Hosmer–Lemeshow test result of χ^2^ = 7.966 (*P* = 0.336), a maximum Youden index of 0.701, an optimal cutoff value of 2.207, sensitivity of 77.7%, and specificity of 92.3%. Calibration curve analysis showed that the nomogram's predictions closely matched the ideal outcomes. Decision curve analysis (DCA) demonstrated that when the threshold probability exceeded 0.1, the nomogram's clinical net benefit surpassed those of the "full intervention" and "no intervention" strategies, highlighting its strong clinical applicability. Additionally, MSTN was positively correlated with CRP and Hs-CRP, while GHRL was negatively correlated with MSTN, CRP, and Hs-CRP. Significant differences were observed between MSTN, GHRL, and CRP (*P* < 0.05).

**Conclusion:**

This study supports the hypothesis that age, right upper limb diameter, simplified anorexia scale score, and MSTN are significant risk factors for malnutrition in CHF patients. The nomogram developed in this study demonstrated robust predictive value for identifying malnutrition in this population. Furthermore, the proposed inflammation-GHRL/MSTN-appetite improvement/muscle growth-CHF improvement pathway offers a potential regulatory mechanism,which represents a promising direction for research into the mechanisms of malnutrition and muscle loss disorders in patients with CHF.

**Supplementary Information:**

The online version contains supplementary material available at 10.1186/s12872-025-04985-1.

## Highlights

• This study is the first in China to integrate clinical biochemical indicators, Ghrelin (GHRL), and Myostatin (MSTN) into a nomogram for assessing malnutrition risk in CHF patients;

• It introduces innovative indicators such as GHRL and MSTN to enhance the accuracy of malnutrition risk prediction in CHF patients;

• This is the first study, to our knowledge, to investigate the correlation between GHRL, MSTN, CRP, and Hs-CRP, and to preliminarily explore the physiological and pathological mechanisms underlying malnutrition risk in CHF patients;

• Age, right upper limb diameter, simplified anorexia scale score, and MSTN were identified as significant risk factors for malnutrition in CHF patients (*P*<0.05). The nomogram demonstrated excellent accuracy, discrimination, and clinical applicability.

**Supplementary Information:**

The online version contains supplementary material available at 10.1186/s12872-025-04985-1.

## Introduction

Chronic heart failure (CHF) represents the terminal stage of various cardiovascular diseases [1]. Globally, the number of CHF patients has reached approximately 64 million [2], with a one-year mortality rate ranging from 17 to 45% [3]. Malnutrition is prevalent among CHF patients and is often linked to factors such as reduced appetite, dysphagia, hyposmia, and alterations in intestinal microbiota, particularly in older individuals [4, 5]. Research indicate that patients with poor nutritional status have a 1.8- to 2.5-fold higher risk of mortality compared to those with normal nutritional status [6]. The prevalence of comorbid malnutrition varies significantly among CHF patients, ranging from 10.5% to 90% [7–9]. Sarcopenia is another frequent comorbidity in CHF patients, affecting approximately 34% to 41% of this population [10, 11]. Clinical studies have shown that sarcopenia independently predicts the risk of death in critically ill patients [11, 12] and is strongly associated with clinical problems such as falls [13], fractures [14], and osteoporosis [15].

Currently, there is no universally accepted “gold standard” for assessing the nutritional status of CHF patients. Existing clinical nutritional assessment scales are often complex, time-consuming, and may be influenced by the edema status of CHF patients, limiting their accuracy in reflecting overall nutritional status. For sarcopenia assessment, the 2019 Asian Working Group on Sarcopenia (AWGS) recommended using dual-energy X-ray absorptiometry (DXA) or bioelectrical impedance analysis (BIA) to measure appendicular lean body mass [16]. However, these methods have limited clinical applicability due to their high cost and complex procedures. Therefore, there is a huge unmet need for a simpler and more reliable screening tool for malnutrition in CHF patients.

Growth hormone-releasing peptide (Ghrelin, GHRL) and muscle-specific inhibitory factor (Myostatin, MSTN) are emerging biomarkers with distinct roles in physiological regulation. GHRL promotes appetite by binding to specific hypothalamic receptors that regulate gastric emptying and intestinal motility. Notably, it is the only known centrally mediated peripheral hormone involved in appetite regulation [17]. Beyond its effects on appetite, GHRL plays a key role in various physiological processes, such as enhancing endothelial and vascular function, preventing atherosclerosis, inhibiting cardiac remodeling, and regulating anti-inflammatory, anti-apoptotic, and lipid metabolism pathways [18, 19]. In contrast, MSTN has been implicated as a causative agent of muscle atrophy [20]. Clinical inhibition of the MSTN/activin-ActRIIB pathway can prevent or reverse the loss of muscle mass and strength [21]. Several studies have shown a significant association between nutritional status and muscle loss; poorer nutritional status can trigger an inflammatory response in the body, accelerating muscle catabolism [22]. Despite these insights, no studies have simultaneously examined the interconnected roles of chronic heart failure (CHF), malnutrition, and sarcopenia, leaving a critical gap in understanding their intrinsic links.

A nomogram is a scoring method that assigns weights to influencing factors based on their contribution to the outcome variable in a predictive model. The predictive value of the outcome event is calculated by summing the scores of all variables. Due to its intuitive and comprehensible nature, column line plots have been widely used in clinical practice and are regarded as a relatively reliable predictive tool [23–25]. This study aims to develop a risk prediction model using a column line plot by analyzing the risk factors associated with malnutrition in CHF patients, providing clinicians with a simpler and more reliable screening tool for malnutrition. Additionally, this study will compare the correlations between GHRL, MSTN, C-reactive protein (CRP), and high-sensitivity C-reactive protein (Hs-CRP), to further elucidate the potential relationship between malnutrition/muscle wasting disease and inflammatory pathophysiological mechanisms.

## Materials and methods

### Patients

This study was a prospective cross-sectional observational investigation involving 128 patients with congestive heart failure (CHF) who were admitted to the Department of Cardiology at Guang’anmen Hospital, China Academy of Traditional Chinese Medicine, from February 2022 to February 2023. Among these, 107 patients had CHF combined with malnutrition, while 21 patients had CHF without malnutrition. The inclusion criteria were: ① meeting the diagnostic criteria for CHF [26, 27]; ②NYHA cardiac function classification II-IV; ③patients who provided informed consent. The exclusion criteria included: ① chronic wasting diseases, such as tumors, infections, trauma, and anemia; ② primary liver diseases, including but not limited to cirrhosis and various types of hepatitis associated with hypoproteinemia; ③ chronic renal insufficiency with eGFR < 30% or primary renal diseases resulting in significant proteinuria; ④ severe primary digestive diseases, eating difficulties, and prolonged chronic diarrhea that may affect nutritional status; ⑤ participation in other clinical trials within the preceding two weeks; ⑥ pregnant or lactating women, or individuals with known drug allergies or hypersensitivity; ⑦ psychiatric disorders.

Two groups of patients were divided into malnutrition group and non-malnutrition group based on whether they met the diagnostic criteria for malnutrition, as shown in the Mini Nutritional Assessment -Short Form(MNA-SF) score. The total score was 14 points, with a score > 11 indicating good nutritional status and a score ≤ 11 indicating malnutrition (see Supplementary Table 1) [28]. The study was approved by the Ethics Review Committee (Review No. 2021-KY-019), and all enrolled patients provided informed consent.

### Data collection

Baseline data for hospitalized patients were gathered within 24 h of admission, including name, age, gender, education, vital signs (heart rate, oxygen saturation, body temperature, respiration), family history, duration of disease, treatment course, past medical history, previous oral medications, and comorbidities. Measurements included patients’ heights, weights, body mass index (BMI), abdominal circumferences, diameters of the upper limbs, systolic blood pressures, and diastolic blood pressures. Concurrently, patients’ physicochemical index were collected, including whole blood cytometric analysis + C-reactive protein (CRP), N-terminal prohormone of brain natriuretic peptide (NT-proBNP). Other parameters assessed included NT-proBNP, high-sensitivity CRP (Hs-CRP), creatinine (Cr), blood urea nitrogen (BUN), glucose (Glu), total cholesterol (TC), triglycerides (TG), low-density lipoprotein (LDL), high-density lipoprotein (HDL), very-low-density lipoprotein (VLDL), hemoglobin, albumin, pre-albumin, total proteins, and ferritin. The assessment of patients’ cardiac function included NYHA classification, Minnesota Quality of Life Score, MNA-SF scale score, Simple Anorexia Scale score, and Simple Appetite Scale score. Echocardiographic parameters measured included left ventricular ejection fraction (LVEF), with a normal range of 50% to 60%, and left ventricular end-diastolic internal diameter (LVEDD), with normal ranges of < 55 cm for men and < 50 cm for women.

### Statistical analysis

Statistical analyses were performed using SPSS version 25.0 and R programming language. Measurement data were presented as mean ± standard deviation (X ± S). T-tests were employed for normally distributed measurement data, while rank-sum tests were used for non-normally distributed data. Count data were expressed as percentages, and the Chi-square (χ^2^) test was conducted. Correlation analyses utilized Pearson correlation analysis for normally distributed data; otherwise, Spearman correlation analysis was applied. Risk factors for malnutrition in CHF patients were determined using multifactorial logistic regression analysis, and a predictive model for malnutrition risk was developed using the “rms” package in R. Internal validation was conducted using receiver operating characteristic (ROC) curves and calibration curves to assess the model’s differentiation and accuracy, while the Hosmer–Lemeshow test evaluated the model’s goodness of fit. Decision curve analysis (DCA) was employed to assess the clinical utility of the model. A *p*-value of < 0.05 was considered statistically significant.

## Research results

### Comparison of clinical baseline data

In this study, 128 patients with CHF were included, of which 107 were also diagnosed with malnutrition. The results indicated that BMI, body weight, abdominal circumference, albumin, prealbumin, MNA-SF scale score, simple anorexia scale score, and simple appetite scale score were significantly lower in the malnourished group compared to the non-malnourished group (*P* < 0.01). Additionally, the diameter of the right upper limb, hemoglobin, and GHRL levels were significantly lower in the malnourished group than in the non-malnourished group (*P* < 0.05). Conversely, Hs-CRP levels, Minnesota quality of life scores, and MSTN levels were significantly higher in the malnourished group (*P* < 0.05). The age of patients in the malnourished group was significantly greater than that of the non-malnourished group (*P* < 0.01) (see Table 1).

**Table 1:** General baseline characteristics of the two groups of CHF patients

	Malnutrition group	Non-malnutrition group	Z/T/χ^2^	*P*
Age	80 (73,85)	70 (63,79)	−3.184	0.001
Sex (male)	49 (45.8%)	10 (47.6%)	0.024	0.878
BMI	21.83 ± 4.79	25.45 ± 3.83	10.67	0.001
Educational (≥ middle school)	81 (76.42%)	16 (80%)	6.65	0.476
Height	165 (158,170)	164 (162.5,165.5)	−0.34	0.735
Weight	57 (51,67)	66.5 (60.5,75.5)	−3.57	0.001
Systolic pressure	128.99 ± 20.55	135.24 ± 17.79	−1.3	0.412
Diastolic pressure	70.53 ± 13.93	78 ± 8.678	−2.364	0.056
Heart rate	72 (64,80)	76 (65.5,83)	−1.111	0.267
Finger oxygen	96 (94,98)	96 (95,97.5)	−0.019	0.984
Temperature	36.4 (36.2,36.5)	36.3 (36.2,36.5)	−1.258	0.208
Breathe	18 (18,20)	18 (18,20)	−0.399	0.692
NT-proBNP	4600 (1932.5,953.5)	3583.5 (1352,10,215.5)	−0.56	0.583
LVEDD	47 (44,53)	49 (43,55.5)	−0.26	0.801
LVEF	55 (43,59)	53 (44.5,60)	−0.6	0.550
CRP	5.00 (1.23,18.43)	2.96 (0.5,9.67)	−1.641	0.101
Hs-CRP	5.98 (1.96,21.59)	2.47 (1.14,7.85)	−2.278	0.023
Minnesota Quality of Life Rating	76 (67,84)	68 (51,77)	−2.58	0.010
MNA-SF scale scores	7 (5,8)	12 (11.5,13)	−7.26	< 0.001
Simple Anorexia Scale Score	7 (6,8)	9 (8,10)	−5.05	< 0.001
Simplified Appetite Scale Score	10 (8,12)	13 (11,15)	−4.174	< 0.001
Cr	89 (66,108)	81 (76.5,88.5)	0.08	0.780
BUN	6.75 (6.45,8.43)	7.19 (6.78,7.63)	0.20	0.660
Glu	5.87 (5.32,6.90)	5.64 (4.46,6.96)	−0.145	0.885
Abdominal Circumference	87.42 ± 13.57	97.34 ± 9.51	7.80	0.006
Left upper limb diameter	24.20 ± 4.02	24.38 ± 6.53	0.02	0.880
Right upper limb diameter	23.53 ± 3.54	25.69 ± 2.57	4.38	0.040
TC	3.77 ± 1.24	3.71 ± 1.26	0.04	0.835
TG	1.13 ± 0.63	1.31 ± 0.56	1.47	0.230
HDL	1.05 (0.89,1.69)	1 (0.75,1.40)	2.88	0.090
LDL	2.33 ± 0.83	0.35 ± 0.96	0.007	0.930
VLDL	0.52 ± 2.30	0.60 ± 0.25	1.32	0.245
Hemoglobin	112.97 ± 24.13	126.33 ± 19.17	5.72	0.020
Albumin	33.90 ± 5.56	37.06 ± 3.31	6.34	0.010
Total protein	63.62 ± 13.77	64.13 ± 6.03	0.028	0.872
Ferroprotein	126.88 (44.95,238.78)	152.72 (65.77,240.69)	−0.07	0.945
Pre-albumin	14.96 ± 7.21	20.12 ± 4.53	9.96	0.002
MSTN	3015.58 (1915.41,4942.93)	2469.14 (1132.54,3545.67)	−1.98	0.047
GHRL	259.26 (111.11,645.95)	452.99 (209.87,998.12)	−2.26	0.024

*P* < 0.05 indicates significant difference

*P* < 0.01 indicates highly significant difference

### Multivariate logistic regression

Univariate analysis identified 15 significantly different indicators: BMI, age, weight, Hs-CRP, Minnesota quality of life score, MNA-SF scale score, simplified anorexia scale score, simplified appetite scale score, abdominal circumference, right upper limb diameter, hemoglobin, albumin, pre-albumin, MSTN, and GHRL. A multivariate logistic regression analysis was performed with malnutrition as the dependent variable and these 15 indicators as covariates. The Omnibus test for model coefficients yielded *P* < 0.05, indicating successful model fitting. The Hosmer–Lemeshow test showed *P* = 0.990 > 0.05, indicating excellent model building and simulation fit. The results showed that the simplified anorexia scale score (0-no appetite, 10-normal appetite) was a protective factor for CHF patients with malnutrition (OR = 0.282, *P* = 0.009 < 0.05). MSTN (OR = 1.001, *P* = 0.021 < 0.05), age (OR = 1.124, *P* = 0.013 < 0.05), and right upper limb diameter (OR = 1.809, *P* = 0.026 < 0.05) were risk factors for CHF patients with malnutrition (See Table 2).

**Table 2:** Logistic regression analysis of risk factors for malnutrition in CHF patients

	β	SE	Wald	OR	95% confidence interval		*P* value
					Lower limit	Upper limit	
Age	0.116	0.047	6.106	1.124	1.024	1.232	0.013
Weight	−0.045	0.089	0.253	0.956	0.804	1.138	0.615
Right upper limb diameter	0.593	0.266	4.983	1.809	1.075	3.044	0.026
Simple Anorexia Scale Score	−1.267	0.484	6.859	0.282	0.109	0.727	0.009
Simplified Appetite Scale Score	−0.094	0.265	0.127	0.91	0.542	1.529	0.722
MSTN	0.001	0	5.303	1.001	1	1.002	0.021
GHRL	0	0.001	0.04	1	0.997	1.002	0.842
Albumin	−0.089	0.17	0.271	0.915	0.656	1.277	0.603
BMI	−0.2	0.256	0.614	0.819	0.496	1.351	0.433
Hemoglobin	−0.045	0.024	3.397	0.956	0.911	1.003	0.065
Minnesota Quality of Life	0.05	0.029	3.05	1.051	0.994	1.112	0.081
Hs-CRP	0.052	0.068	0.596	1.054	0.923	1.203	0.44
Pre-albumin	−0.164	0.116	1.988	0.849	0.675	1.066	0.159

*P* < 0.05 indicates significant difference

*P* < 0.01 indicates highly significant difference

### Construction and validation of a nomogram

**Fig. 1:**
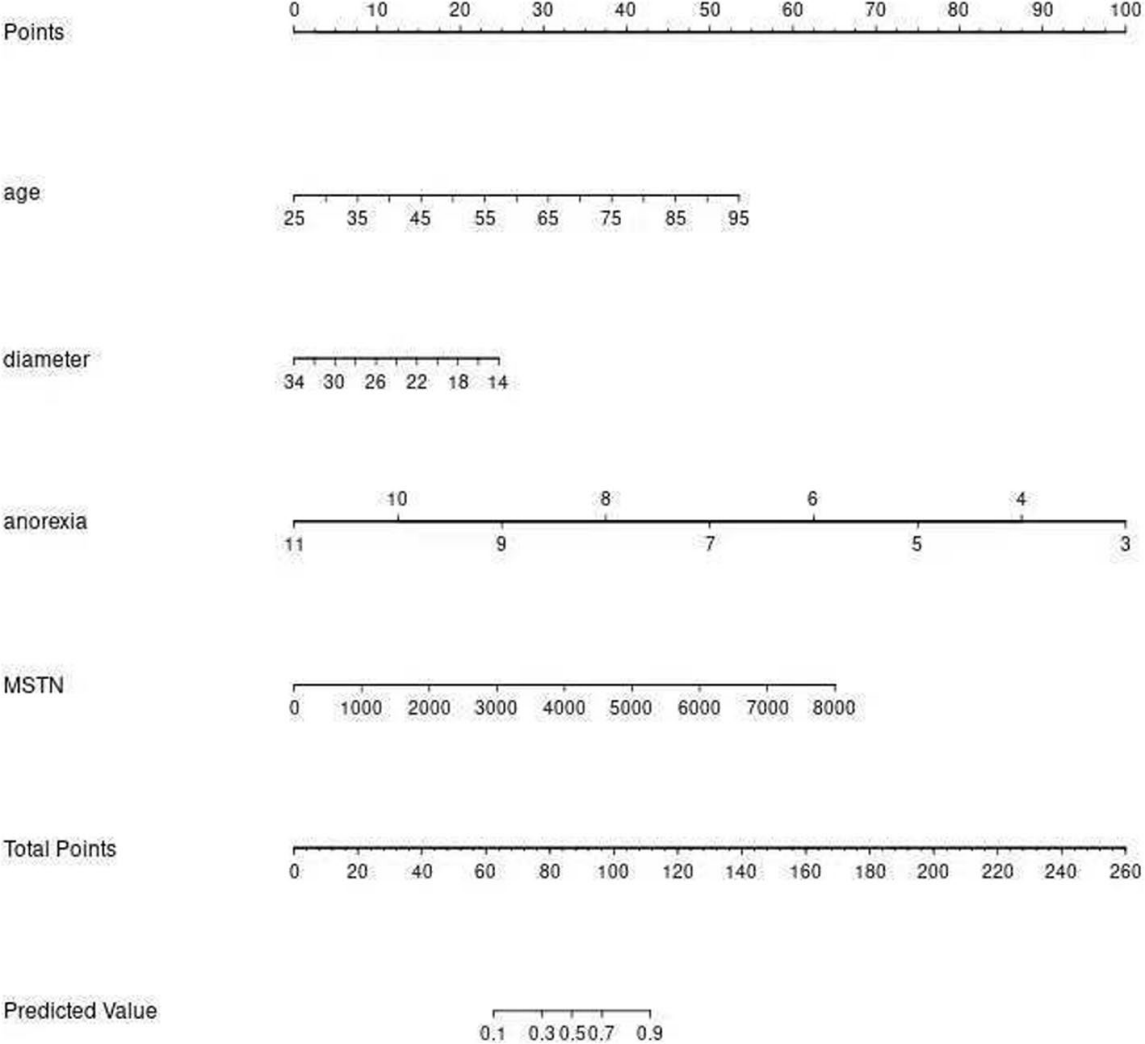
Developed prediction nomogram

**Fig. 2:**
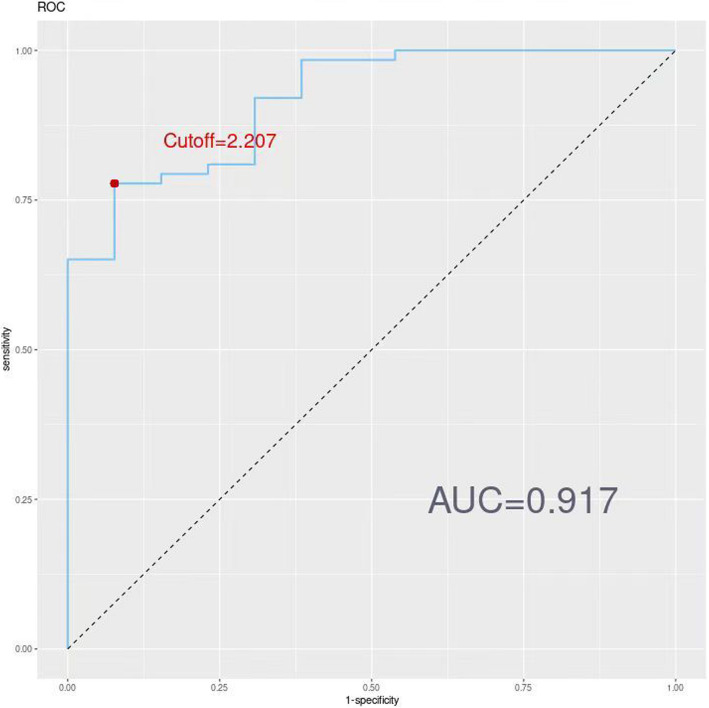
The ROC curves of the nomogram

**Fig. 3:**
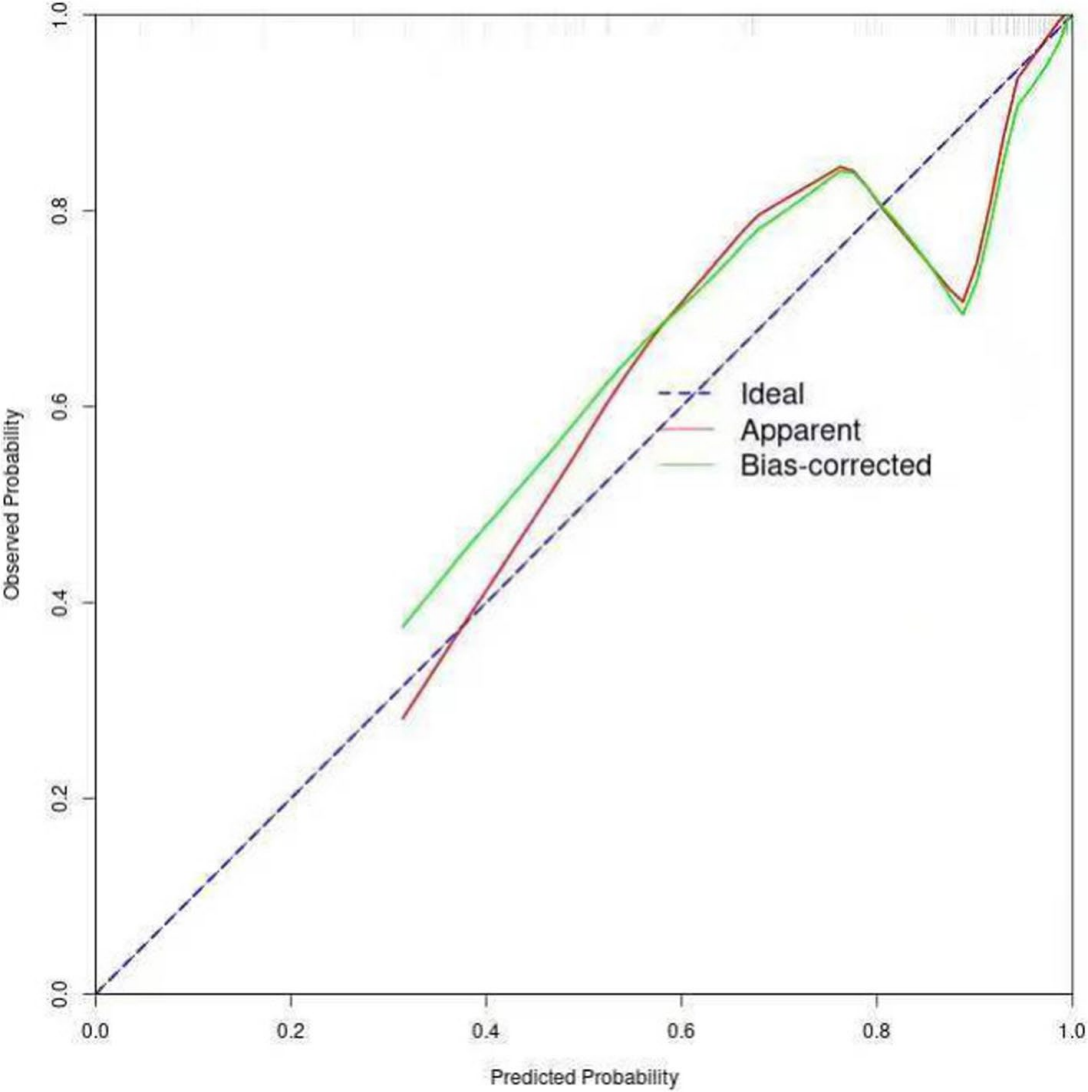
Calibration curves

**Fig. 4:**
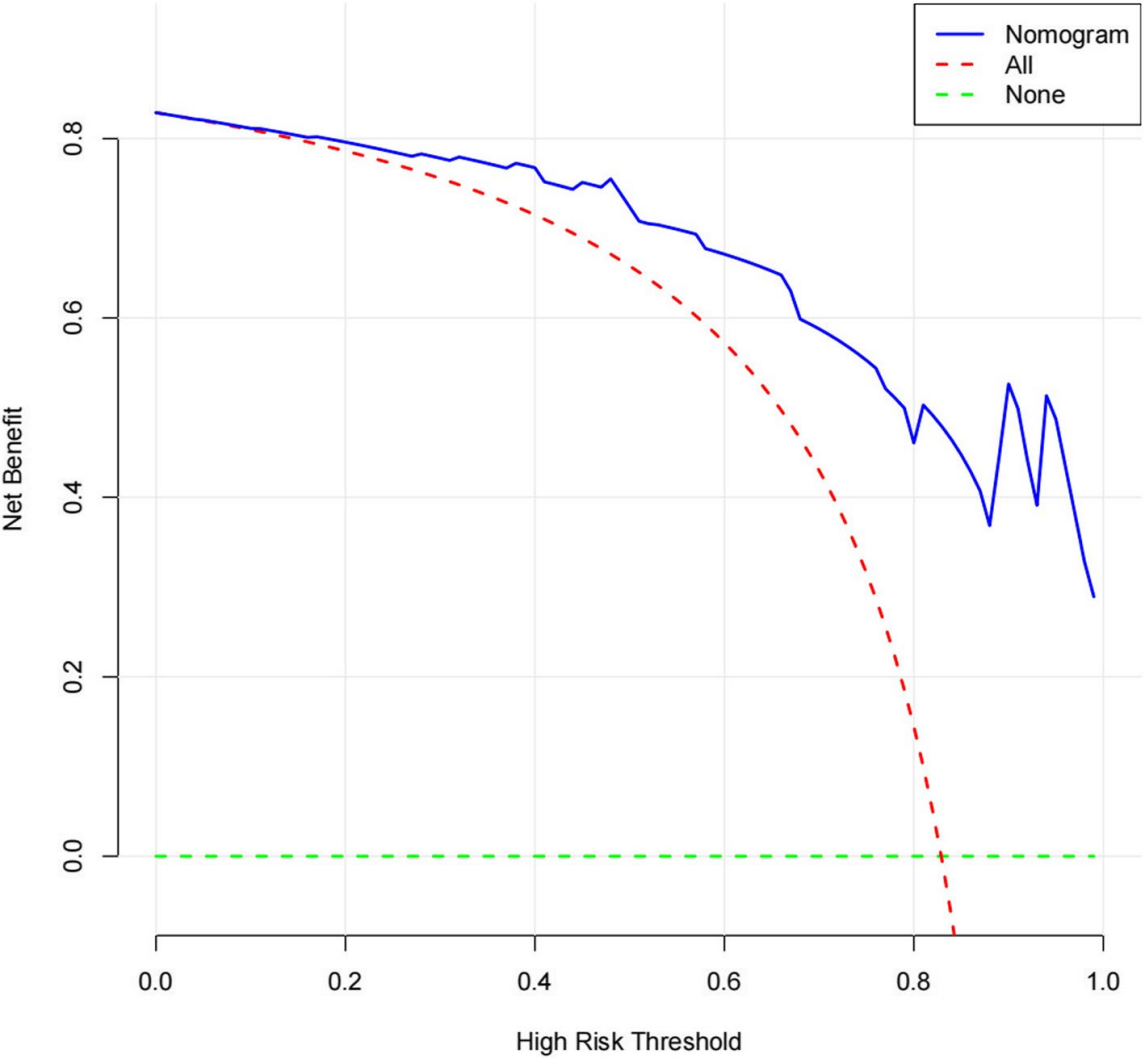
Decision curve analysis (DCA)

The four independent variables—age, right upper limb diameter, simplified anorexia scale, and MSTN—showed significant differences (*P* < 0.05) in the multifactorial regression analysis and were used as predictors to construct a nomogram (Fig. 1). The nomogram exhibited strong discriminative power during internal validation, with an AUC of 0.917 (95% CI: 0.8439–0.990), a Hosmer–Lemeshow test result of χ^2^ = 7.966 (P = 0.336), a maximum Youden index of 0.701, an optimal cutoff value of 2.207, sensitivity of 77.7%, and specificity of 92.3% (Fig. 2). Calibration curve analysis showed that the nomogram's predictions closely matched the ideal outcomes (Fig. 3). Decision curve analysis (DCA) demonstrated that when the threshold probability exceeded 0.1, the nomogram’s clinical net benefit surpassed those of the"full intervention"and"no intervention"strategies, highlighting its strong clinical applicability (Fig. 4).

### Correlation analysis between GHRL, MSTN, CRP, Hs-CRP

In clinical practice, we further revealed the relationship between GHRL, MSTN, CRP, and Hs -CRP through correlation analysis, aiming to further elucidate the potential pathophysiological mechanisms between malnutrition/sarcopenia and inflammation (see Table 3).

**Table 3:** Correlation analysis between GHRL, MSTN, CRP, Hs-CRP

Dimensions	MSTN	GHRL	CRP	Hs-CRP
MSTN	1			
GHRL	−0.045	1		
CRP	0.189	−0.189	1	
Hs-CRP	0.077	−0.161	0.65	1

The study demonstrated a positive correlation between MSTN and CRP, Hs-CRP, and a significant difference between MSTN and CRP (*P* = 0.038 < 0.05). Conversely, a negative correlation was observed between GHRL and MSTN, CRP, Hs-CRP, and a significant difference between GHRL and CRP (*P* = 0.039 < 0.05).

## Discussion

Malnutrition and sarcopenia are common comorbidities in patients with CHF [18], and the combination of CHF with malnutrition often leads to several negative consequences, including poor wound healing, decreased immunity, and a diminished quality of life [29]. In addition, malnutrition-induced hypo-osmolarity often exacerbates edema in patients with CHF [30]. Clinical studies have demonstrated that nutritional interventions can improve the overall health of patients suffering from malnutrition and sarcopenia [15, 31]. In our study, age, right upper extremity diameter, Simple Anorexia Scale score, and MSTN were identified as risk factors for the development of malnutrition in patients with CHF, By integrating the four aspects of age, physicochemical indexes, morphological indexes, and scale scores, we developed a relatively comprehensive and reliable prediction model. The validity of the nomogram was demonstrated based on AUC, calibration curves, DCA curves, and the Hosmer–Lemeshow test. The variables included in this nomogram can provide an intuitive and convenient reference for clinical decision-making by clinical healthcare professionals, so as to provide accurate treatment plans for patients [32].

Strength, endurance, and nutritional status in older adults typically decline with age. Relevant studies have proposed that the structure of gut flora in the elderly changes with age, evidenced by a decrease in the abundance of gut microbiota genera with anti-inflammatory effects and an increase in the abundance of gut microbiota genera with pro-inflammatory effects. This shift suggests that age-related changes in gut microorganisms often affect intestinal permeability, leading to reduced nutrient absorption, decreased secretion of gastric acid, and diminished protective effects of the intestinal mucosa [33, 34]. In this study, we found that the risk of malnutrition increased by 0.124 times for each additional year of age, highlighting age as a significant predictor of malnutrition in CHF patients.

MSTN is a negative regulator of muscle mass, primarily expressed in skeletal muscle, and is a key contributor of muscle atrophy [21]. Activation of the MSTN/activin signaling pathway plays a critical role in triggering accelerated muscle catabolism [22]. In this study, MSTN levels were higher in the CHF with malnutrition compared to those without malnutrition. We found that the risk of malnutrition increased by 0.001-fold for every 1 pg/ml increase in MSTN, indicating that muscle growth was more significantly inhibited in the malnutrition group. This suggests that the subcutaneous muscles may be relatively weaker, reflecting the nutritional status of the organism to some extent. Malnutrition and sarcopenia often co-exist in clinical practice. In 2012, Vandewoude et al. proposed the “malnutrition-sarcopenia syndrome (MSS),” defined as “a clinical manifestation of malnutrition and age-related accelerated loss of lean body mass, strength, and/or physical function” [35]. Our study found that “the diameter of the right upper limb was smaller in the malnourished group than in the non-malnourished group” and that “the risk of malnutrition increased by 0.809 times for every 1-cm decrease in the diameter of the right upper limb.” These findings further support the association between muscle loss and malnutrition in CHF patients. This is consistent with clinical studies showing that decreased muscle strength combined with malnutrition leads to prolonged hospitalization [36].

GHRL is primarily produced by cells in the stomach, intestines, and hypothalamus, and exerts its pro-appetite effects through its interaction with specific hypothalamic receptors [18]. Randomized controlled trials (RCTs) have demonstrated that GHRL agonists significantly accelerate gastric emptying and increase body weight in women with anorexia nervosa [37]. Additionally, clinical studies indicate that high plasma GHRL levels serve as a positive prognostic indicator in chronic heart failure (CHF), with plasma GHRL negatively correlated with NT-proBNP levels and positively correlated with left ventricular ejection fraction (LVEF) [38]. GHRL-specific receptors are present in cardiomyocytes, where GHRL enhances endothelial and vascular function, prevents atherosclerosis, and inhibits cardiac remodeling [39]. In isolated skeletal muscle, studies have shown that GHRL resists lipolysis, stimulates fat oxidation, and protects muscle from insulin resistance under high fatty acid concentrations [40]. Given its effects on skeletal muscle and appetite, GHRL is considered a potential intervention to improve the nutritional status of CHF patients. In this study, we found that for each 1-point increase in the simple anorexia scale score, the risk in the malnourished group was 0.282 times higher than in the non-malnourished group, suggesting that decreased appetite is associated with poorer nutrition in CHF patients. Furthermore, MSTN was negatively correlated with GHRL in the correlation analysis, implying that improving appetite could enhance the overall nutritional status of CHF patients. Clinical studies have found that duodenal infusion of nutrient solution reduces GHRL release by 65% in lean individuals compared to obese individuals, who are more sensitive to hunger and satiety signals [41]. Therefore, caregivers should monitor the appetite of CHF patients and adjust the type and mode of nutritional support promptly to achieve better prognostic outcomes.

CHF is characterized by chronic low-level inflammation, with significant changes in innate immune cytokines and chemokines, such as a marked increase in tumor necrosis factor (TNF-α) and interleukin-6 (IL-6) [42]. In the later stages of CHF, some patients may develop cardiac cachexia, which is often associated with a more severe inflammatory state that depletes the body’s nutrients, leading to hypoproteinemia, anemia, and osteoporosis. Some studies suggest that more attention should be paid to the “malnutrition-inflammation complex syndrome” [35]. In this study, a positive correlation was observed between MSTN and CRP, indicating that greater muscle loss is associated with higher CRP levels. Conversely, there was a negative correlation between GHRL and CRP, suggesting that increased appetite is correlates with lower CRP levels. GHRL and MSTN are involved in various inflammatory metabolic pathways in the human body [43–47]. Therefore, controlling inflammation may be an effective therapeutic target. Current clinical studies have shown that enteral nutrition therapy in elderly CHF patients not only improves overall nutritional status but also reduces the expression of inflammatory factors [48]. This study proposes that the inflammation-GHRL/MSTN-appetite improvement/muscle growth-CHF improvement pathway may be a key regulatory mechanism in the development of malnutrition and muscle loss in CHF patients,which represents a promising direction for research into the mechanisms of malnutrition and muscle loss disorders in patients with CHF.

## Limitations

(i) The sample size in this study is limited, with a relatively small number of participants. Future multi-center studies with larger sample sizes and external validation are needed to further assess the generalizability of the model.
(ii) This study identified a correlation among GHRL, MSTN, CRP, and Hs-CRP based on clinical observations; however, the level of evidence remains relatively weak, and potential confounding variables cannot be excluded. Future research should involve more standardized and reliable large-scale randomized controlled trials to eliminate confounding factors and enhance statistical power, thereby allowing for a more accurate understanding of the causal relationships between GHRL, MSTN, CRP, and Hs-CRP.
(iii) The sample size included in this study is relatively small. Although the results of the model have been interpreted with caution, it is important to acknowledge that the model’s complexity and multicollinearity among predictive factors may impact the results. Therefore, expanding the sample size in future studies is necessary.

## Supplementary Information

Supplementary Material 1: Table S1. Diagnostic criteria for malnutrition

## Data Availability

The authors confirm that the data supporting the findings of this study are available within the article.
