# Supplementary material for: A nomogram for predicting malnutrition risk in patients with chronic heart failure and correlation study between GHRL, MSTN, CRP, Hs-CRP
Source: BMC Cardiovasc Disord. 2025 Aug 14;25:603. doi: 10.1186/s12872-025-04985-1 (PMC12351787; doi:10.1186/s12872-025-04985-1)
Supplement: Supplementary file 1 — Supplementary Material 1: Table S1. Diagnostic criteria for malnutrition [file 12872_2025_4985_MOESM1_ESM.docx]

***Supplementary***

**Table 1** Diagnostic criteria for malnutrition

| Index | Score | | | |
| --- | --- | --- | --- | --- |
| Weight loss in the past three months | ＞3kg 0 point | Unclear 1 point | 1-3kg 2 points | NO 3 points |
| BMI | ＜19 0 point | 19-21 1 point | 21-23 2 points | ＞23 3 points |
| Stress or acute illness in the past three months | No 0 point | Yes 2 points |  |  |
| Activity ability | Bedridden 0 point | Able to move, unwilling to 1 point | Can go out for activities 2points |  |
| Mental illness | Severe dementia and depression 0 point | Mild dementia 1 point | No 2 points |  |
| Loss of appetite, dyspepsia, difficulty chewing and swallowing in the last 3 months | Severe loss of appetite  0 point | Mild loss of appetite 1point | Without these symptoms 2points |  |
